# Temporal and morphological impact of pressure overload in transgenic FHC mice

**DOI:** 10.3389/fphys.2013.00205

**Published:** 2013-08-27

**Authors:** Hao Chen, Hyosook Hwang, Laurel A. K. McKee, Jessica N. Perez, Jessica A. Regan, Eleni Constantopoulos, Bonnie LaFleur, John P. Konhilas

**Affiliations:** ^1^Molecular Cardiovascular Research Program, Department of Physiology, University of ArizonaTucson, AZ, USA; ^2^Mel and Enid Zuckerman College of Public Health, University of ArizonaTucson, AZ, USA

**Keywords:** pressure overload, cardiac hypertrophy, concentric hypertrophy, remodeling, myocardial relaxation, familial hypertrophic cardiomyopathy

## Abstract

Although familial hypertrophic cardiomyopathy (FHC) is characterized as cardiac disease in the absence of overt stressors, disease penetrance, and pathological progression largely depend on modifying factors. Accordingly, pressure overload by transverse aortic constriction (TAC) was induced in 2-month-old, male mice with and without a FHC (R403Q) mutation in α-myosin heavy chain. A significantly greater number of FHC mice (*n* = 8) than wild-type (WT) mice (*n* = 5) died during the 9-week study period. TAC induced a significant increase in cardiac mass whether measured at 2 or 9 weeks post-TAC in both WT and FHC mice, albeit to a different extent. However, the temporal and morphological trajectory of ventricular remodeling was impacted by the FHC transgene. Both WT and FHC hearts responded to TAC with an early (2 weeks post-TAC) and significant augmentation of the relative wall thickness (RWT) indicative of concentric hypertrophy. By 9 weeks post-TAC, RWT decreased in WT hearts (eccentric hypertrophy) but remained elevated in FHC hearts. WT hearts following TAC demonstrated enhanced cardiac function as measured by the end-systolic pressure-volume relationship, pre-load recruitable stroke work (PRSW), and myocardial relaxation indicative of compensatory hypertrophy. Similarly, TAC induced differential histological and cellular remodeling; TAC reduced expression of the sarcoplasmic reticulum Ca^2+^-ATPase (2a) (SERCA2a; 2 and 9 weeks) and phospholamban (PLN; 2 weeks) but increased PLN phosphorylation (2 weeks) and β-myosin heavy chain (β-MyHC; 9 weeks) in WT hearts. FHC-TAC hearts showed increased β-MyHC (2 and 9 weeks) and a late (9 weeks) decrease in PLN expression concomitant with a significant increase in PLN phosphorylation. We conclude that FHC hearts respond to TAC induced pressure overload with increased premature death, severe concentric hypertrophy, and a differential ability to undergo morphological, functional, or cellular remodeling compared to WT hearts.

## Introduction

Familial hypertrophic cardiomyopathy (FHC) is clinically heterogeneous; some individuals experience limited or no clinical symptoms while subgroups of patients develop severe deterioration of cardiac function and symptoms of congestive heart failure (CHF; Maron, 2002). The clinical diagnosis of FHC is one of exclusion, hypertrophy in the absence of an overt cardiac disease etiology. Consequently, identification of FHC is based on the presence of left ventricular hypertrophy (LVH) plus a mutation in a gene typically encoding sarcomeric proteins (Bonne et al., 1998; Towbin, 2000; Marian and Roberts, 2001). Yet, the severity of these underlying abnormalities especially LV geometry correlates with the risk for sudden cardiac death (Assayag et al., 1997). Consequently, phenotypic expression of FHC largely depends on the interaction of the specific mutation and modifying factors such as genetics, lifestyle, environment, and other co-existing diseases (Lechin et al., 1995; Bonne et al., 1998; Osterop et al., 1998). For example, approximately half of patients that harbor a missense mutation at residue 403 (R403Q) of β-myosin heavy chain (β-MyHC) die by age 45 from sudden cardiac death or CHF (Geisterfer-Lowrance et al., 1990; Epstein et al., 1992) whereas others experience much milder symptoms. Accordingly, identifying underlying etiologies responsible for the clinical heterogeneity of FHC becomes tantamount to initiating an appropriate treatment strategy. Therefore, it is critical to identify the cellular and molecular pathology resulting from the interaction of FHC and these modifying factors.

Arterial hypertension (high-blood pressure) results in cardiac pressure overload coupled with LVH and is the most common medical problem in the US (Collins et al., 1990; Kannel, 1996; Ong et al., 2007). Much like LVH due to FHC, a critical predictor of clinical prognosis is the geometric pattern of LVH (Devereux et al., 1994). Considering the clear variability in the patterning of LVH geometry in the clinical population, it is likely that there is a significant inheritable component. In support of this contention, C57BL/6J (B6) and 129S1/SvImJ subjected to pressure overload display distinct patterns of LVH illustrating the differential susceptibility based solely on genetic background (Barrick et al., 2007).

Given the prominence of hypertension, it has been suggested that a significant number of patients with unidentified FHC have hypertension (20%), contributing to its variable penetrance (Vinereanu et al., 2001; Ortlepp et al., 2002). Interestingly, when this is modeled in mice with an FHC (Arg403Gln) mutation in the native α-cardiac myosin heavy chain (MHC) gene (α MHC^403/+^), the response to pressure overload, not surprisingly, depends on genetic background. Following pressure overload, α MHC^403/+^ mice on the 129SvEv background show no significant differences from wild-type (WT) controls whereas α MHC^403/+^ mice on the outbred Black Swiss (BS) background show worsening LV geometry, mass, and function compared to WT controls (Schmitt et al., 2003). This suggests that even in the context of an inheritable FHC mutation, genetic background plays a prominent role in the phenotypic manifestation of this disease.

Because FHC symptoms often develop progressively over time during the development of the disease, the purpose of this study is 2-fold: (1) to further investigate the impact of pressure overload on FHC disease pathogenesis using an alternative FHC mouse model harboring the same mutation (R403Q) but on the C57Bl6 background, and (2) to investigate the temporal impact of pressure overload on the early course of FHC disease pathogenesis. Because the FHC mice in this study develop early clinical signs of FHC (Vikstrom et al., 1996), we hypothesized that FHC mice will respond to pressure overload with worsening cardiac pathology more similar to the α MHC^403/+^ mice on the BS background. In this study, we induced gradual (progressive) pressure overload in WT and FHC mice by applying a loose band to the transverse aorta of young mice (2 months of age) and allowing them to mature concurrently with increasing afterload. We subsequently examined 2 separate experimental groups at 2 and 9 weeks post-transverse aortic constriction (TAC) for cardiac function, ventricular morphology, and cellular remodeling. Here, we show that WT and FHC mice develop significant cardiac hypertrophy in response to pressure overload but that FHC mice show signs of worsening pathology through earlier pathological onset, premature death, severe concentric hypertrophy, and attenuated ability to adapt at the functional and cellular level. We conclude that co-existing pressure overload and genetic background play a modifying role in the early course of FHC disease pathogenesis.

## Materials and methods

### Animal models

The experimental murine model has been detailed previously and consisted of male mice heterozygous for the mutant α-myosin transgene (Vikstrom et al., 1996). The transgene coding region contains a point mutation, R403Q, and a deletion of 59 amino acids in the actin-binding site bridged by the addition of 9 non-myosin amino acids. WT littermates were used as controls for the FHC mice. All experiments were performed using protocols that adhered to guidelines and approved by the Institutional Animal Care and Use Committee at the University of Arizona, and to 2011 NIH guidelines for care and use of laboratory animals.

### Transverse aortic constriction (TAC)

To determine the temporal impact of TAC on cardiac function, ventricular morphology, and cellular remodeling, separate experimental groups of mice were analyzed 2 and 9 weeks post-TAC. Furthermore, because male FHC mice develop clinical signs of pathology [myocyte hypertrophy and disarray and expression of fetal genes (Vikstrom et al., 1996)] by 4 months of age, TAC surgery was performed at 2 months of age, prior to overt pathology. Mice were anesthetized by intraperitoneal injection of Avertin (2,2,2 Tribromoethanol dissolved in 2-methyl-2-butanol, 0.5 g/kg, Sigma). The animals were placed in a supine position, and the chest was shaved using a chemical hair remover. The aortic arch was exposed through the 1st intercostals. A 6-0 nylon suture was placed between the brachiocephalic trunk and the left common carotid artery. A blunted 26-gauge needle was placed against the aorta, and the aorta was tightened along with the needle. We chose to use a 26-gauge needle, because a previous study had shown that aortic banding to a 27-gauge stenosis induced moderate hypertrophy without clinical signs of decompensated hypertrophy at 5 weeks after aortic banding (Hill et al., 2000). By using the bigger size of needle, we intended to produce even more moderate and gradual hypertrophic responses, which we believe is more clinically relevant. The needle was removed and the chest was closed. In sham-operated mice, the aorta was isolated but not tied. The mice were allowed to recover on a temperature-controlled heating pad until fully recovered.

### Echocardiography

Two and 9 weeks post-TAC, echocardiography was carried out to non-invasively examine morphological and functional changes associated with pressure overload by TAC. Transthoracic echocardiography was performed using a Visual Sonics Vevo 770 high-resolution imaging system (Visual Sonics, Toronto, ON, Canada). A 25-MHz transducer was used. The chests of animals were shaved with a chemical hair remover. Esthesia was maintained by 1% isoflurane with oxygen. Body temperature was maintained using a heated platform. Respiratory rates and electrocardiograms were monitored throughout the study.

Two-dimensional M-mode echocardiographic images were obtained from the parasternal short-axis views at the level of the mid-ventricles. Cardiac chamber dimensions and the left ventricular wall thickness were measured. Interventricular septum (IVS), left ventricular posterior wall thickness (LVPWT), and internal dimension (LVIDd) were measured from the M-mode images. Relative wall thickness (RWT) [(LVPWT/LVIDd) × 2] was calculated from the M-mode measurements. Diastolic function was assessed by conventional pulsed-wave Doppler analysis of mitral valve inflow patterns from the apical four chamber views. Data was analyzed off-line using Vevo 770 analytic software. The data were obtained in triplicate and averaged.

### Hemodynamics and measurements of pressure-volume loop relationships

*In vivo* hemodynamics and pressure-volume loop relationships were studied. Anesthesia was maintained by an intraperitoneal injection of avertin (0.5 g/kg). Endotracheal intubation was performed. Animals were ventilated (145 breaths/minute and 0.2 ml tidal volume). A closed chest approach was utilized. A 1.4-French pressure-volume catheter (Scisense, London, ON, Canada) was inserted into the left ventricle via catheterization of the right carotid artery. The position of the catheter was carefully adjusted until stable pressure-volume loops were obtained. The catheter was connected to a signal processor (Scisense, London, ON, Canada). Data were obtained digitally using iWorx data acquisition/analysis system (iWorx Systems, Dover, NH). Tracings of left ventricular pressure and volume were collected at a sampling rate of 1000 Hz. The volume was calibrated in each mouse by the calculation of parallel conductance. Parallel conductance is a measure of the contribution of the ventricular wall to the volume signal and is determined by the time-varying electrical conductance (Porterfield et al., 2009). The parallel conductance measured was subtracted from the volume signal to yield an absolute volume.

The end-systolic and end-diastolic pressure-volume relationships were studied by a transient inferior vena cava (IVC) occlusion. Briefly, the abdomen was opened, and the IVC located between liver and diaphragm was identified. The transient venous occlusion was carried out by pinching-off the vena cava using a pair of plastic forceps. Immediately after measurements of pressure-volume loops, mice were killed by cervical dislocation. The heart was removed and cut into 2 halves. One half was fixed with phosphate-buffered paraformaldehyde (4%) overnight (4° C) and processed for histological analysis. The other half was snap-frozen with liquid N_2_ and stored (−80° C) for biochemical analysis.

### Functional parameters

The following parameters were recorded and analyzed: heart rate (HR), LV end systolic and diastolic pressure (ESP and EDP, respectively), end systolic and diastolic volume (ESV and EDV, respectively), the maximal rates of LV pressure increase and decrease (dp/dtmax and dp/dtmin), and time constant of LV pressure decay (tau). The hemodynamic data at baseline were measured for 3 s immediately before the induction of a transient IVC occlusion and averaged. Left ventricular pressure-volume relations were evaluated from pressure-volume loops recorded during transient occlusion of the IVC. Pre-load recruitable stroke work (PRSW), end-systolic pressure-volume relationship (ESPVR) and the slope (Ees), end-diastolic pressure-volume relationship (EDPVR) and the slope (stiffness constant), and dp/dtmax and EDV relationship (dp/dtmax—EDV) were calculated using iWorx systems software.

### Picrosirius red (PSR) staining and determination of collagen content

The paraformaldehyde fixed tissue was prepared using a tissue processor and embedded in paraffin. The embedded tissue was sectioned in 7 μ m thickness. The specimens were dewaxed, rehydrated, and stained with PSR to detect collagen fibers. The PSR stained tissue specimens were photographed with a camera connected to a polarized light microscope (ZEISS Axio Imager M1) to detect birefringence of collagen fibers. Three fields were chosen randomly from each heart sample. The images were quantified by a semi-automated imaging analysis program (AxioVision). A color threshold was defined in such a way to detect mature collagen. The area of birefringence was normalized by the total area of interest.

### Sample preparation and expression of myosin heavy chain isoform (MyHC)

MyHC isoforms were studied using SDS-PAGE as previously described (Konhilas et al., 2006). Snap frozen ventricular tissue was homogenized (Next Advance, Averill Park, NY) in 8 M Urea buffer (Urea 8M, Thiourea 2M, Tris 0.05 M, DTT 75 mM, SDS 3%, Bromophenol blue 0.05%, pH 6.8). Samples were analyzed using SDS-PAGE (6% acrylamide, 37.5:1 cross-linked with DATD). Gels were run in a Se600 Hoefer gel system at 4° C at a constant current of 16 mA per gel. The gels were fixed and stained using a Silver Stain Plus kit (BioRad 161-0449), dried, and scanned (EPSON V750 PRO). The ratio of α-MyHC and β-MyHC isoforms expressed was determined using densitometry (LabImage 1D L340 N3, Kaplean BioImaging System, Leipzig, Germany). In order to confirm MyHC staining was within linear densitometric range, six or seven dilutions of each sample were analyzed covering a 50–60 fold dilution range. Soleus muscle which harbors β-MyHC was used as a β-MyHC standard. From the linear relationships determined for each MyHC isoform, the relative MyHC content of each isoform was extrapolated and compared to the densitometric values obtained.

### Western blot analysis

Frozen heart samples were prepared as detailed above and run using 10% SDS polyacrylamide gel electrophoresis (SDS-PAGE) and transferred to a PVDF membrane. The transferred proteins were incubated with anti-phospholamban (PLN, Thermo Scientific), anti-phospho-S16-phospholamban (p-PLN, Upstate), sarcoplasmic reticulum Ca^2+^ ATPase (SERCA2A, Thermo Scientific), total-cTnI (cTnI, AbCam), cTnI-ser^23/24^ (cTnI-S^23/24^, Cell Signaling) primary antibodies, then incubated with horseradish-conjugated secondary antibodies. The enzymatic activity was detected using enhanced chemiluminescent substrate kits. The intensity of the protein band was determined using densitometry (LabImage 1D L340 N3, Kaplean BioImaging System, Leipzig, Germany). In addition, prior to immunoblotting, all membranes were stained with Ponceau S acid red and quantified for total protein. Next, total protein measured by coomassie blue and/or Ponceau S was compared to MHC expression for equal loading. All hearts of a given genotype at both 2 and 9 week timepoints were analyzed on a single immunoblot. All immunoblot analysis was performed from the semi-quantitation of individual blots and was not compared across blots according to the guidelines set forth by the American Physiological Society.

### Statistical analysis

Data are presented as Mean ± SE. Mean differences among different groups were examined using 2-way analysis of variance (SPSS, IBM, version 19). *Post-hoc* tests were carried out using Bonferroni *t*-tests. The logrank test was performed to compare the survival distributions of WT TAC and FHC TAC. *P*-values < 0.05 were considered to be significant.

## Results

### Survival

**Figure 1:**
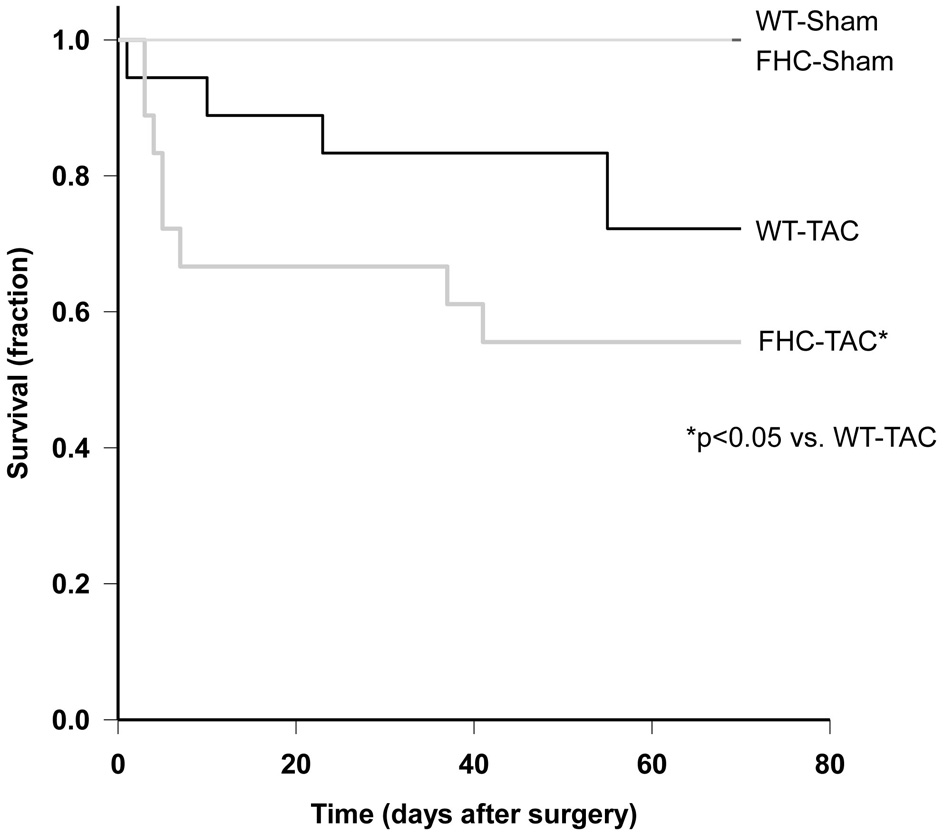
**Kaplan–Meier survival analysis of WT and FHC mice after TAC.** Animals subjected to pressure overload died over the period of the study. Mortality rate was greater in the mutant animals compared to WT mice (*p* < 0.05).

For the 9-week timepoint, a total of 58 mice (WT *n* = 29, FHC *n* = 29) received either TAC (WT *n* = 18, FHC *n* = 18) or sham procedure (WT *n* = 11, FHC *n* = 11). None of the sham animals died during the study period, however, TAC surgery resulted in a statistically significant mortality rate where 13 of 36 (36%) died. Gross anatomical exam of the expired mice revealed no significant changes in cardiac-pulmonary system and no significant anatomical changes in cardiac morphology. Of the mice that underwent TAC surgery, those harboring the FHC mutant transgene demonstrated greater mortality (8 of 18; 44%) during the entirety of the study period, compared to WT mice (5 of 18; 28%). A Kaplan–Meier curve summarizes these findings (Figure 1).

### Cardiac hypertrophy and echocardiography

**Figure 2:**
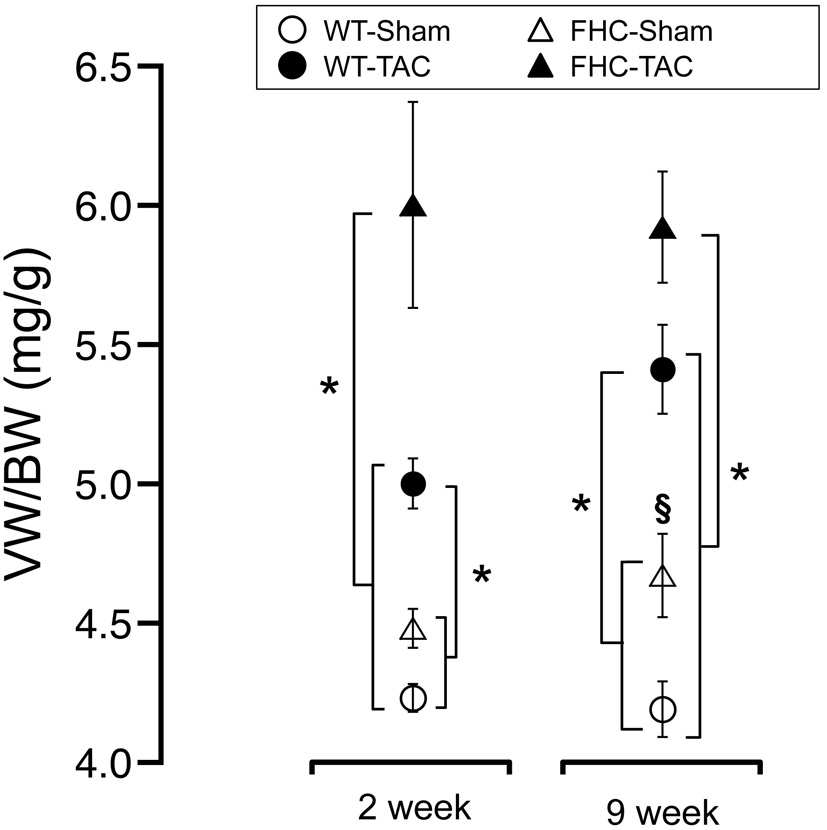
**Summary of normalized cardiac mass in WT and FHC mice with and without TAC.** VW/BW at the 2- and 9-week timepoint was determined by dividing ventricular mass (in mg) by body weight (in g). Data presented as mean ± s.e.m. Experimental group numbers are indicated in Table 1. (^*^p < 0.05; ^§^*p* < 0.05 from values obtained in WT-Sham).

We determined the impact of the FHC mutation and TAC on cardiac hypertrophy through absolute and normalized heart weight. The morphometric data from this study are summarized in Figure 2 and Table 1. Consistent with previous data, at the 2-week timepoint (Stauffer et al., 2006), the presence of the FHC mutation in sham mice did not result in a measurable hypertrophy by ventricular weight and ventricular weight normalized to body weight (BW; Figure 2) or tibial length (Table 1). Following 2-weeks of TAC, both WT and FHC mice developed significant hypertrophy. Although FHC hearts following TAC were larger by absolute or normalized heart mass, the proportional increase (26.8 ± 5.7 vs. 18.7 ± 3.4% in FHC compared to WT hearts, respectively) in cardiac mass was not statistically significant.

**Table 1:** **Body and cardiac mass**.

	**WT-Sham (*n* = 10)**	**WT-TAC (*n* = 12)**	**FHC-Sham (*n* = 11)**	**FHC-TAC (*n* = 10)**
**2 WEEKS POST-TAC**				
**Morphometry**				
BW, g	23.4 ± 0.5	23.5 ± 0.7	23.4 ± 0.6	22.3 ± 0.5
Tibial length (mm)	18.4 ± 0.1	17.8 ± 0.2	18.5 ± 0.1	18.4 ± 0.1
VW (mg)	99.2 ± 1.7	117.7± 5.9^1^	105.0 ± 3.0^3^	133.1 ± 5.2^1^^,^^2^^,^^3^
VW/TL (mg/mm)	5.4 ± 0.1	6.6 ± 0.2^1^	5.7 ± 0.2^3^	7.4 ± 0.3^1^^,^^2^^,^^3^
**9 WEEKS POST-TAC**				
**Morphometry**				
BW, g	26.7 ± 0.9	27.7 ± 0.8	27.1 ± 1.0	27.3 ± 0.8
Tibial length (mm)	20.4 ± 0.2	20.1 ± 0.2	20.1 ± 0.2	20.3 ± 0.2
VW (mg)	98.8 ± 3.1	133.8 ± 5.9^1^	126.4 ± 4.7^2^^,^^3^	161.4 ± 6.8^1^^,^^2^^,^^3^
VW/TL (mg/mm)	4.8 ± 0.1	6.5 ± 0.2^1^	6.2 ± 0.2^2^^,^^3^	7.2 ± 0.3^1^^,^^2^^,^^3^

*Summary of morphometric data from WT and FHC mice with and without TAC at 2 and 9 weeks post-TAC. VW/TL was determined by dividing cardiac mass (in mg) by tibial length (in mm) values are presented as Mean ± s.e.m*.

1 *p < 0.05 compared to corresponding sham controls*.

2 *p < 0.05 compared to corresponding WT animals*.

3 *p < 0.05 compared to WT-TAC animals. BW, body weight; VW, ventricular weight. Numbers in parentheses indicate the number of animals per group*.

Previous work has shown that male FHC mice develop measurable hypertrophy by 4 months of age (Vikstrom et al., 1996; Olsson et al., 2001). Similarly, at the 9-week timepoint (4 months of age), FHC-Sham hearts were larger than WT-Sham hearts indicative of cardiac hypertrophy (Figure 2 and Table 1). In response to 9 weeks of pressure overload by TAC, both WT and FHC mice developed significant cardiac hypertrophy indicated by an increase of 35.1 ± 6.2 and 30.6 ± 4.2% in ventricular weight, respectively, over corresponding sham controls (Table 1). Ventricular weight normalized to BW or TL showed a similar increase. This robust hypertrophic response was observed despite the use of a larger gauge (26 gauge) needle in order to induce a more gradual pressure overload (see Methods). The extent of the hypertrophic response, although proportionally greater in FHC than in WT mice, again, was not statistically different between the two groups.

**Figure 3:**
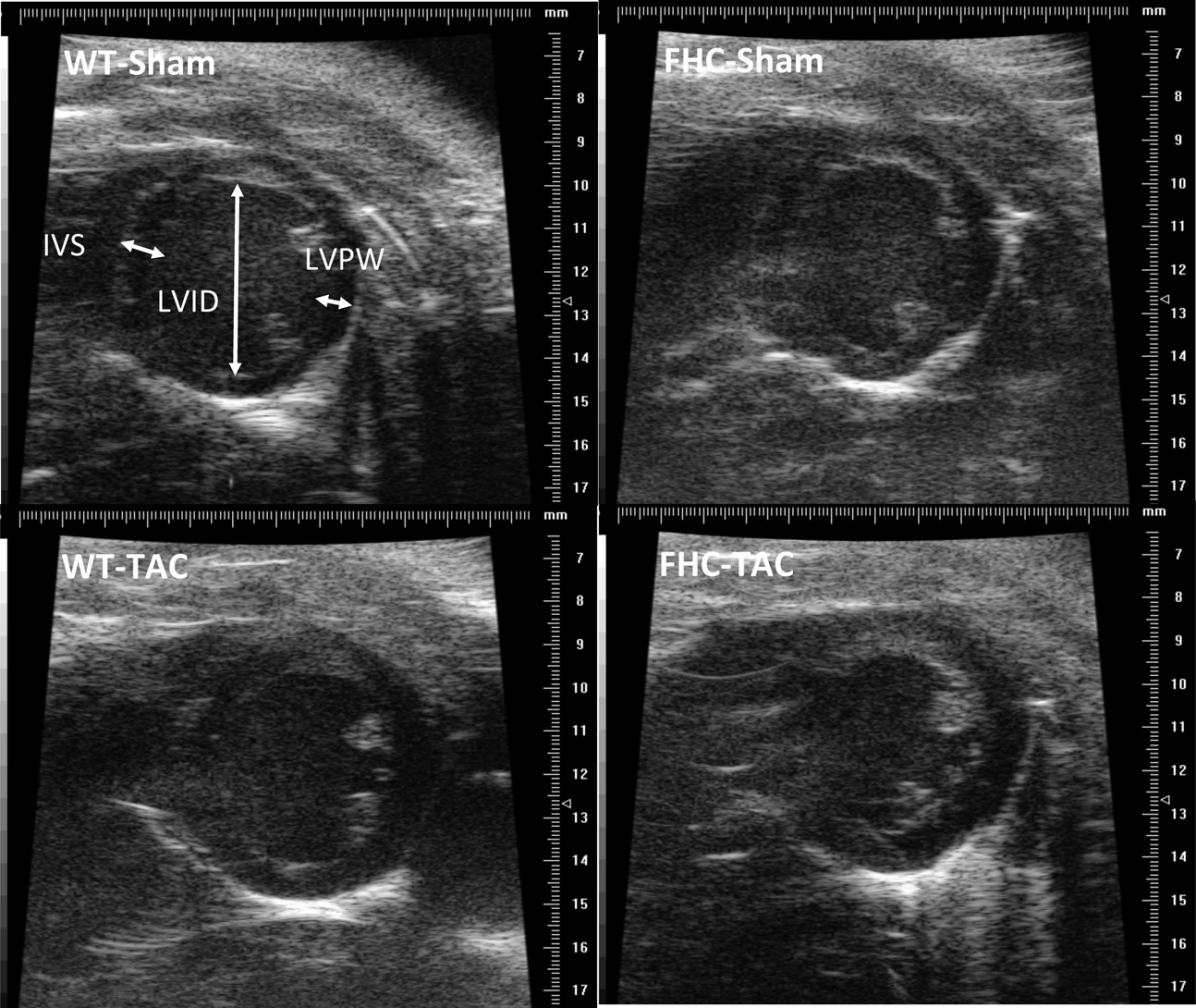
**Representative 2-dimentional echo images showing left ventricular chamber from the parasternal short-axis views.** From M-mode images, morphological measurements were made and parameters were calculated as detailed in the Methods. Pressure overload resulted in an increase in cardiac mass and wall thickness without appreciable changes in chamber dimension. The extent of increase in wall thickness is significantly greater in the FHC-TAC mice compared to WT-TAC mice.

**Figure 4:**
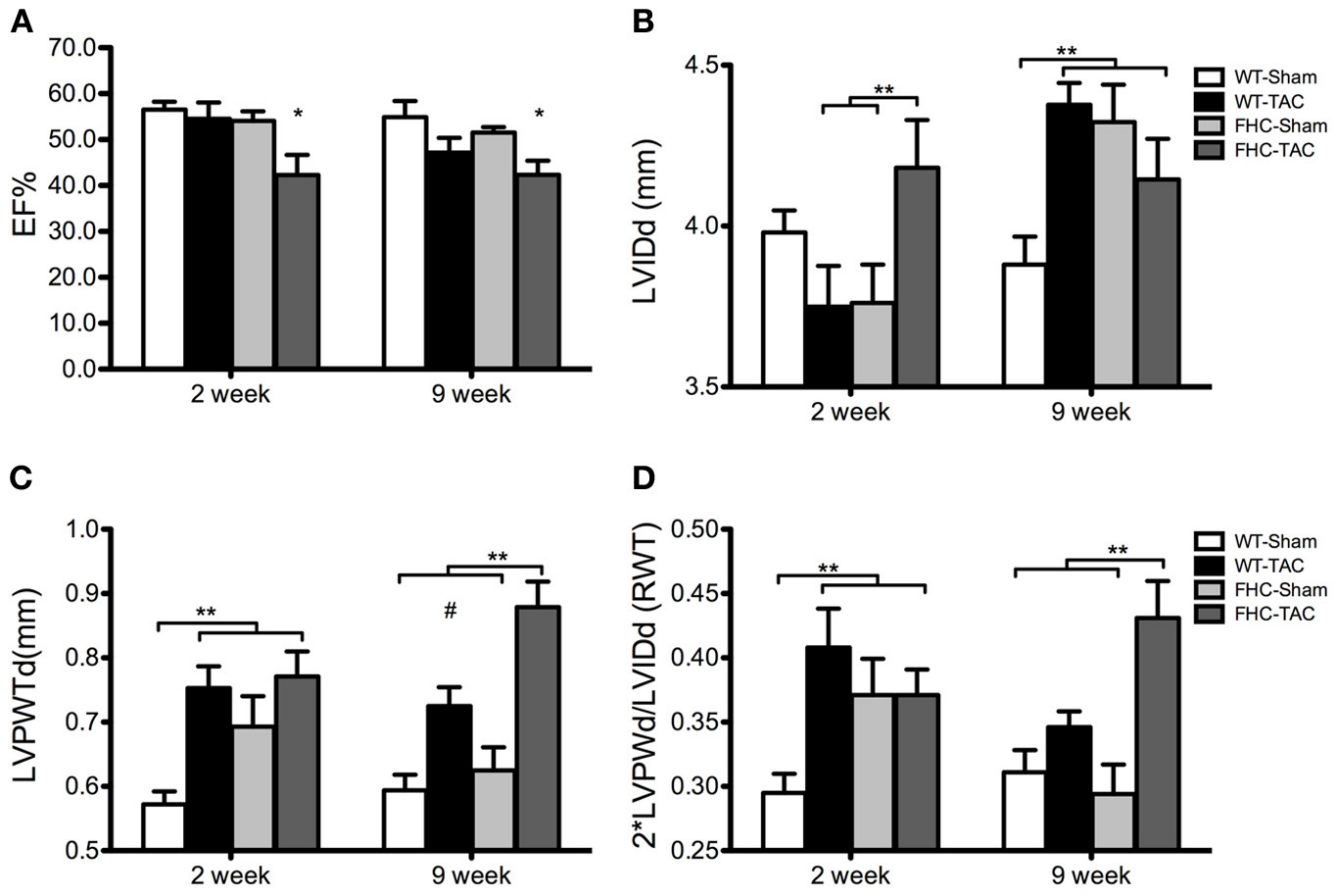
**Echocardiographic parameters of ventricular function and morphometry in WT and FHC mice with and without TAC. (A)** Ejection fraction (EF%); **(B)** LVIDd is LV internal diameter at end-diastole; **(C)** LVPWTd is LV posterior wall thickness at end-diastole; **(D)** RWT is relative wall thickness calculated as 2^*^LVPWTd/LVIDd and represents a measure of LV eccentricity. Data presented as mean ± s.e.m. Experimental group numbers are as follows, 2 week group: WT-Sham, *n* = 9; WT-TAC, *n* = 8; FHC-Sham, *n* = 7; FHC-TAC, *n* = 8. For 9 week group: WT-Sham, *n* = 9; WT-TAC, *n* = 10; FHC-Sham, *n* = 9; FHC-TAC, *n* = 8. (^*^p < 0.05 from values obtained in WT-Sham; ^**^p < 0.05; ^#^*p* < 0.05 from values obtained in WT-Sham and FHC-Sham).

To assess the impact of pressure overload by TAC on cardiac function and *in situ* ventricular morphometry, high-resolution 2-deminsional echocardiography was performed on WT and FHC-Sham and -TAC mice at each timepoint. Representative B-mode images are illustrated in Figure 3. A functional decline as a result of pressure overload by TAC was observed only in FHC mice at the early (2 week) timepoint compared to all other groups (Figure 4A). This functional deficit persisted in FHC mice following TAC at the late (9 week) timepoint only when compared to WT-Sham mice; there was no longer a difference between WT-TAC, FHC-Sham and FHC-TAC mice.

Although TAC induced cardiac hypertrophy in both WT and FHC mice, the regional hypertrophic impact was different between the 2 groups. The early (2 weeks post-TAC) response of FHC hearts was a significant increase in left-ventricular internal dimension during diastole (LVIDd) compared to FHC-Sham hearts whereas WT hearts responded to pressure overload with a slight decrease that was not different from WT-Sham hearts but reached significance when compared to FHC hearts following TAC (Figure 4B). By 9 weeks of TAC, the LVIDd was greatest in both FHC and WT hearts compared to WT-Sham hearts. Interestingly, FHC-Sham hearts developed a progressively larger LVIDd, which was significantly greater than WT-Sham hearts but not compared to both TAC groups.

Despite a similar LVIDd between both sham groups at the 2 week timepoint, FHC-Sham hearts showed an increased thickness in the LV posterior wall during diastole (LVPWTd) compared to WT-Sham hearts (Figure 4C). However, TAC induced a significant thickening of the LVPW during diastole in both groups compared to WT-Shams but similar to FHC-Shams. At 9 weeks post-TAC, LVPWTd continued to increase in FHC-TAC hearts to a thickness that was significantly greater than all other groups. LVPWTd measured in WT-TAC hearts remained significantly greater than WT-Sham counterparts. Interestingly, the LVPWTd of FHC-Sham hearts was no longer different than WT-Sham hearts.

The consequence of these morphometric changes was an early (2 weeks post-TAC) concentric hypertrophic response to TAC as measured by the relative wall thickness (RWT) that persisted in FHC-TAC hearts at 9 weeks post-TAC but progressed to eccentric hypertrophy in the WT-TAC group at 9 weeks post-TAC (Figure 4D). Unlike FHC hearts following TAC, FHC-Sham hearts exhibited a progressively eccentric hypertrophic response.

### Left-ventricular hemodynamics and pressure-volume loop analysis

Considering that the trajectory of ventricular remodeling was impacted by the FHC transgene, we assessed *in vivo* LV hemodynamics at 9 weeks post-TAC and the data are summarized in Table 2. As mentioned above, the hearts of both WT and FHC mice responded to the gradual pressure overload by TAC with significant hypertrophy. As expected, TAC similarly increased systolic pressure in both WT and FHC mice by 43 ± 9.4 and 42 ± 9.4%, respectively. These data demonstrate that WT and FHC hearts were exposed to a similar magnitude of pressure overload. Furthermore, TAC did not alter the maximum time derivative of pressure development (dp/dtmax) but did significantly increase the maximum time derivative of pressure decline (dp/dtmin) and also shortened the time constant of LV pressure fall (tau) in WT but not in FHC mice.

**Table 2:** ***In vivo* indexes of LV hemodynamics of WT and FHC mice with and without TAC at 9 weeks post-TAC**.

**Parameter**	**WT-Sham (*n* = 9)**	**WT-TAC (*n* = 10)**	**FHC-Sham (*n* = 9)**	**FHC-TAC (*n* = 8)**
HR, bpm	425 ± 14	431 ± 11	428 ± 8	442 ± 8
ESP, mmHg	89 ± 3	127 ± 7^1^	85 ± 5	121 ± 2^a^
dp/dtmax, mmHg/s	6363 ± 185	6670 ± 322	6738 ± 465	7104 ± 498
dp/dtmin, mmHg/s	−5276 ± 258	−7670 ± 445^1^	−5252 ± 275	−6553 ± 545
Ees, mmHg/μl	2.2 ± 0.4	3.2 ± 0.3^1^	3.0 ± 0.5	3.5 ± 0.2
PRSW, mmHg	53 ± 5	87 ± 10^1^	59 ± 4	76 ± 8
tau (W), ms	8.2 ± 0.4	6.5 ± 0.2^1^	7.6 ± 0.3	7.3 ± 0.4
Ea, mmHg/μl	3.8 ± 0.3	6.5 ± 1.0^1^	3.7 ± 0.2	7.2 ± 0.4^1^

*Values are presented as Mean ± s.e.m*.

a *p < 0.05 compared to corresponding sham controls. ESV, End-systolic volume; EDV, end-diastolic volume; ESP, end-systolic pressure; EDP, end-diastolic pressure; Ees, end-systolic elastance; PRSW, preload-recruitable stroke work; Ea, arterial elastance*.

**Figure 5:**
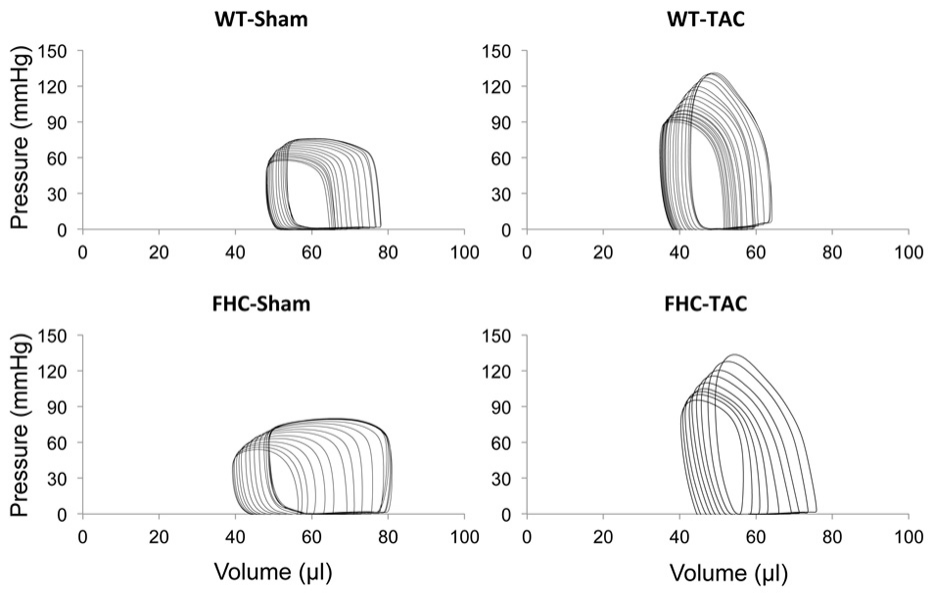
**Four representative LV pressure-volume loop tracings from WT and FHC hearts with and without TAC at 9 weeks post-TAC**.

To determine load-independent parameters of ventricular function (Burkhoff et al., 2005), pressure-volume loops were generated following IVC occlusion. A set of representative pressure-volume loops following IVC occlusion from each experimental group is shown in Figure 5 and summarized in Table 2. Although TAC in WT mice did not elevate dp/dtmax, it significantly enhanced end-systolic elastance (Ees, the slope of the end-systolic pressure-volume relationship; ESPVR), relative to WT-Sham, indicating an increase in contractility. This was also paralleled by an increase in PRSW. Although these parameters were altered by TAC in WT mice, we did not observe a similar change in pressure-overloaded FHC mice. These data indicate that cardiac function was increased in WT-TAC mice, whereas cardiac function was unaltered in FHC-TAC mice, suggesting compensation of cardiac function in WT mice compared to FHC mice in response to TAC. In addition, pressure overload resulted in a similar increase in effective arterial elastance in both groups indicating a similar impact of TAC on arterial load.

### Cardiac remodeling

**Figure 6:**
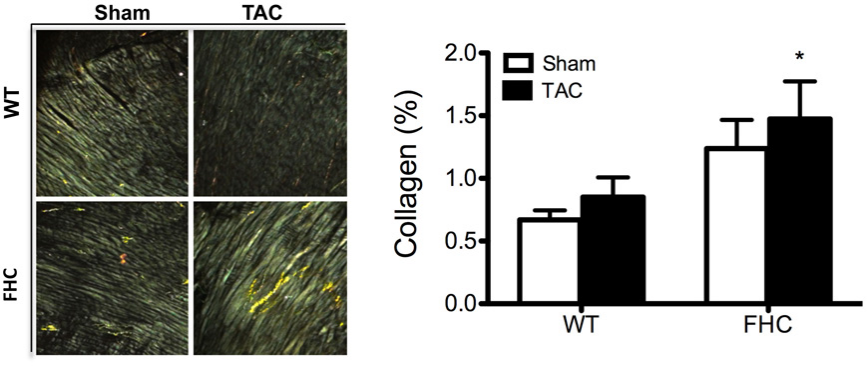
**Collagen deposition in the hearts of WT and FHC mutant mice after 9 weeks pressure overload. Left panel:** Polarized microscopic views of myocardial collagen. **Right panel:** Percentage of fibrosis. Myocardial sections were stained with PSR. Thin collagen fibers have green birefringence and thick collagen fibers have bright yellow/orange birefringence under polarization light microscopy. Birefringence of collagen fibers was quantified using a semi-automated imaging analysis program. ^*^p < 0.05 compared to WT-sham.

**Figure 7:**
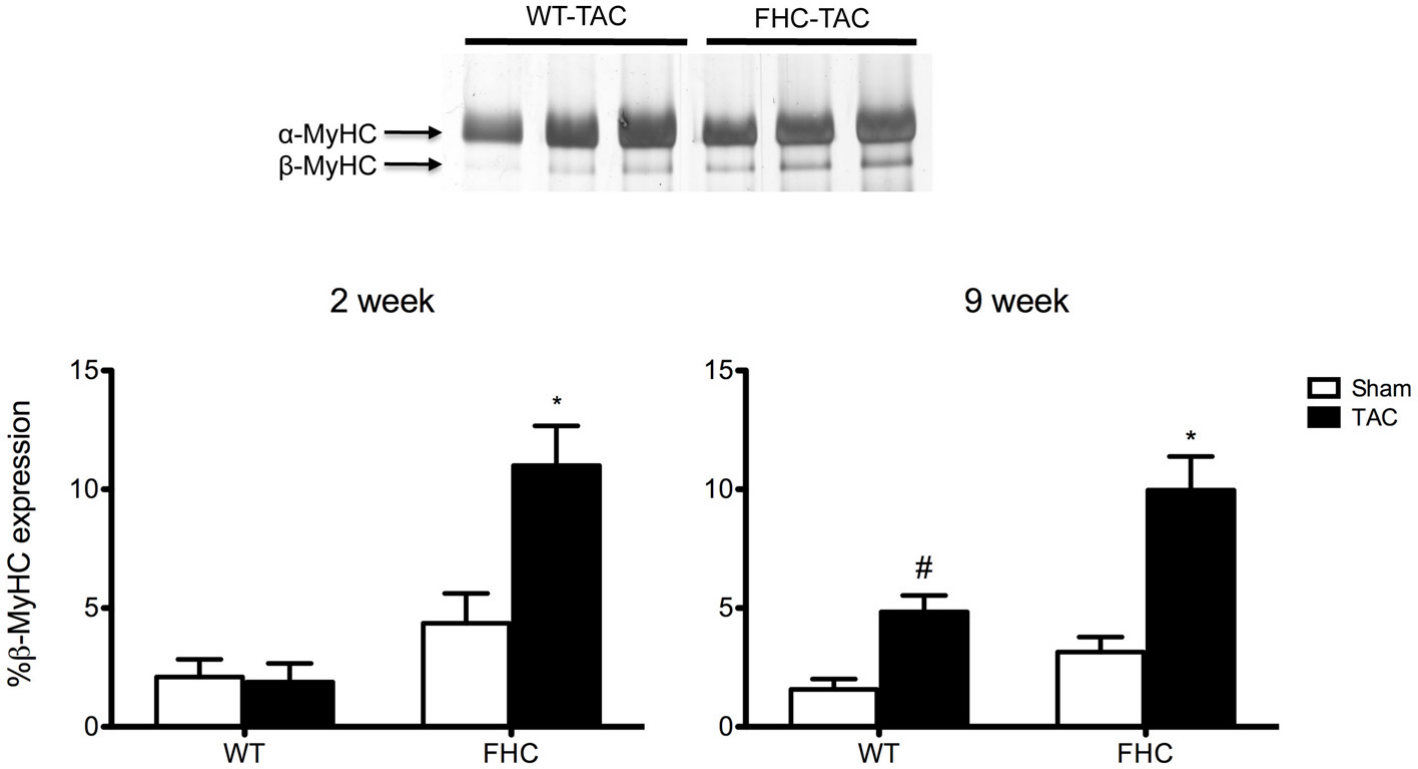
**Expression of β-MyHC protein in WT and FHC mice with and without pressure overload. Top panel:** Representative SDS-PAGE following silver staining shows separation of α-MyHC and β-MyHC in dilutions of 2-week WT-TAC and FHC-TAC hearts. **Bottom panel:** The expression of β-MyHC increases in response to pressure overload at the 2-week timepoint only in the presence of the FHC mutation. Nine-week TAC causes a significant increase in β-MyHC expression in both WT and FHC mice. ^*^p < 0.01 vs. WT-sham and WT-TAC and HCM-sham; ^#^*p* < 0.05 vs. WT-sham.

In response to pathological stimuli including pressure overload, the heart undergoes remodeling at the structural/ultrastructural, cellular, and genetic level. Illustrative of this remodeling, TAC hearts independent of transgene are enlarged and “stiff” following excision. Hallmarks of this remodeling include increased deposition of collagen resulting in a “stiff” myocardium and a recapitulation of the fetal gene program that includes elevated expression of the β-isoform of myosin heavy chain (β-MyHC; Perrino et al., 2006). Consistent with previous studies (Stauffer et al., 2006), FHC mutant mice exhibited an increased deposition of myocardial collagen (Figure 6). Although FHC hearts showed significantly increased collagen content compared to WT hearts, there was not a measurable increase following TAC in either WT or FHC hearts. Next, we examined whether the R403Q mutation differentially impacts MyHC isoform shift following pressure overload. FHC-TAC hearts were found to have a significantly increased protein expression of β-MyHC at both 2 and 9 weeks post-TAC (Figure 7). Two weeks of pressure overload did not cause a heightened expression of β-MyHC in WT mice, however at the 9 week timepoint WT-TAC mice have significantly higher β-MyHC than sham counterparts (Figure 7).

### Altered expression of Ca^2+^ handling proteins

**Figure 8:**
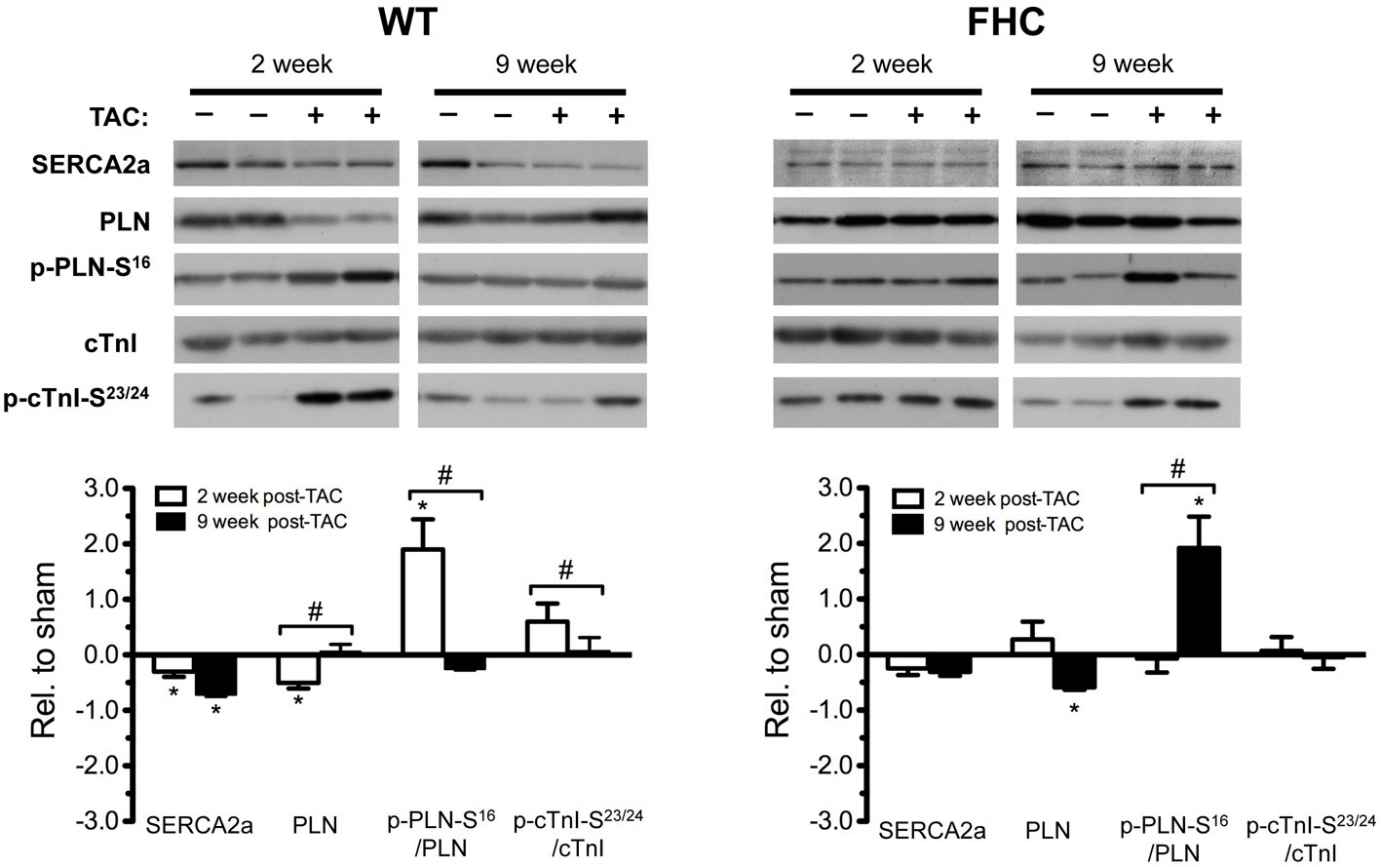
**Expression of Ca^2+^ handling proteins in WT and FHC mice with and without TAC. Top panel:** Ca^2+^ handling proteins, SR Ca^2+^-ATPase (2a) (SERCA2a), phospholamban (PLN), total cTnI, and cTnI-S^23/24^ were assessed by immunoblot analysis. **Bottom panel:** Bar graph representation of the relative impact of TAC in WT and FHC hearts at 2 and 9 weeks post-TAC. In order to better compare the impact of TAC, protein expression in each experimental group subjected to TAC was compared to each respective sham control (*n* = 4 in each group; ^*^p < 0.05 from values obtained in WT-Sham; ^#^*p* < 0.05).

In this study, we observed that TAC induced an increase in load-dependent myocardial relaxation in WT hearts but not in FHC hearts. Diastolic dysfunction is a hallmark of FHC and can result from changes in myofilament function and/or Ca^2+^ handling proteins. We hypothesized that the differential expression and post-translational modification of Ca^2+^ handling proteins underlies these TAC-induced differences in cardiac function. Therefore, we determined protein expression levels of SR Ca^2+^ ATPase (2a) (SERCA2a) and PLN by Western blot analysis in WT and FHC hearts with and without TAC at two different time points (2 weeks and 9 weeks post-surgery) (Figure 8). Pressure overload decreased SERCA2a expression in WT-TAC mice as early as 2 weeks post-TAC and further decreased SERCA2a expression by 9 weeks post-TAC. No significant changes in SERCA2a expression were found at either timepoint in FHC-TAC hearts.

PLN, a regulatory binding partner of SERCA2a, inhibits SERCA2a Ca^2+^ uptake activity when bound. Pathological cardiac stress, such as pressure overload, is known to stimulate the β-adrenergic pathway and subsequently activate a downstream kinase, protein kinase A (PKA; Choi et al., 1997). PKA targets PLN at serine 16 (PLN-S^16^), thus reducing PLN affinity for SERCA, relieving inhibition of SERCA2a activity (Mattiazzi et al., 2005). In WT-TAC hearts, there was an immediate and significant (2 weeks) down regulation of PLN protein levels accompanied by increased PLN-S^16^ phosphorylation (p-PLN-S^16^; Figure 8). The net result was a significant increase in the p-PLN-S^16^/PLN ratio. This became attenuated at 9 weeks post-TAC. On the other hand, early (2 week) TAC in FHC-TAC hearts did not alter PLN expression or altered the level of p-PLN-S^16^ (Figure 8). However, PLN levels in FHC-TAC hearts fell below control levels at 9 weeks post-TAC. Coupled with a slight decrease in total p-PLN-S^16^, there was a significant increase in p-PLN-S^16^/PLN (9 weeks post-TAC).

Desensitization following overstimulation of the β-adrenergic pathway during pathological stress results in a decrease in downstream adrenergic targeting (Choi et al., 1997). Indeed, PKA-dependent phosphorylation of TnI at S22/23 (cTnI-S^23/24^) is reduced in human and animal CHF (McConnell et al., 1997; Kooij et al., 2010). In this study, we determined that the amount of cTnI-S^23/24^ in WT hearts significantly decreased following 9 weeks of TAC compared to 2 weeks. We found that the level of cTnI-S^23/24^ was comparable, regardless of pressure overload in FHC hearts.

## Discussion

In the face of pathological stress such as pressure overload, the heart will adapt in order to meet the prevailing hemodynamic load. Here, we show that cardiac adaptation to pressure overload is characterized by hypertrophy but, more importantly, the pattern of hypertrophy is dependent upon the presence of the mutant myosin FHC transgene. A less obvious conclusion from this study, but perhaps more significant, implicates a more general role for genetic background as a determinant of phenotypic outcome in response to pressure overload. Previous work demonstrates that the type of LVH has a significant inheritable component (Barrick et al., 2007). Here, our findings of pressure overload in FHC mice on the C57/B16 background parallel pressure overload in an alternative R403Q mouse model (α MHC^403/+^) on the BS background but differ from pressure overload in α MHC^403/+^ mice on the 129SvEv background (Schmitt et al., 2003).

It must be noted that although both α MHC^403/+^ and FHC mice were modeled after the R403Q mutation, the mutations were introduced using distinct methodologies: the α MHC^403/+^ mouse model introduced the R403Q mutation into one allele of the α-MHC by homologous recombination, while the FHC mouse model used in this study expressed a mutant α-MHC with expression driven by the rat α-MHC promoter resulting in 10–12% expression of the transgene (Vikstrom et al., 1996). Furthermore, the FHC model in this study consists of a deletion of 59 amino acids in the actin-binding site bridged by the addition of 9 non-myosin amino acids. Because of this unique genetic identity, this study is limited in attributing the phenotypic observations to the R403Q mutation specifically.

Despite the genetic distinctions between the two R403Q mouse models, both models exhibit many of the features seen in humans with FHC, including LV and right ventricular (RV) hypertrophy, cellular disarray, and fibrosis (Geisterfer-Lowrance et al., 1996; Vikstrom et al., 1996). This latter finding holds a particular clinical relevance considering that these two R403Q mouse lines are distinct, pathological FHC models and represent the genetic and phenotypic heterogeneity in the human clinical population. Therefore, these models can be used to elucidate cellular mechanisms leading to the clinical phenotype independent of genotype. Moreover, a recent statement by the National Heart, Lung, and Blood Institute states that, despite a poor mechanistic understanding of this clinical heterogeneity, the overarching goal is to identify novel strategies at preventing the clinical phenotype (Force et al., 2010). It further suggests that success of this goal will largely depend on the delineation of cellular signaling pathways that can modify the FHC phenotype, rather than identification of the specific mutation. Clearly, these two models represent distinct courses of cardiac pathophysiology and underscore the significance of identifying unique modifying factors.

In an attempt to model the clinical development of arterial hypertension in mice, we induced pressure overload by TAC in developing (2-month-old) mice using a less severe aortic constriction (see Methods). This study was also designed to potentially reveal key factors of the pathological trajectory by examining an early (2 weeks post-TAC) and a transitional, pre-CHF (9 weeks post-TAC) timepoint. Although the initial hemodynamic load imparted was presumably lower, both WT and FHC mice show a hypertrophic response measured at 2 and 9 weeks post-TAC. More importantly, the pathological trajectory of the hypertrophic response is very different. FHC-TAC hearts following 2 weeks of TAC are larger than all other groups. However, WT-TAC hearts continue to increase in mass whereas FHC-TAC hearts do not. The suggestion is that FHC hearts reach a hypertrophic “limit” early in the pathological response to TAC compared to WT hearts. Incidentally, FHC-Sham hearts show significant hypertrophy at the 9 weeks (4 months) timepoint consistent with previous findings (Vikstrom et al., 1996; Stauffer et al., 2006).

Along with the increasing cardiac mass, WT-TAC hearts progressively dilate without a change in posterior wall thickness (LVPWd). FHC-TAC hearts, on the other hand, do not dilate but show increased LVPWd thickness. The net result of these morphological changes is an early elevation of the RWT in WT-TAC and FHC-TAC. Once again, the pathological course depends on the presence of the R403Q transgene; at 9 weeks post-TAC, RWT decreases in WT-TAC hearts but continues to increase in FHC-TAC hearts.

In light of these data, the temporal patterning of ventricular structure and geometry in response to pressure overload is dependent on genotype and may lend prognostic value in stratifying patients based on cardiovascular risk as detailed below. Clinical evaluation of hypertensive patients indicates that total mortality correlates with the type of ventricular hypertrophy, or more specifically, ventricular *remodeling* (Koren et al., 1991; Ganau et al., 1992; Gaasch and Zile, 2011). In this study, WT-TAC hearts initially (2 weeks post-TAC) display an elevated RWT with limited hypertrophy and without an increase in chamber dimensions, termed concentric *remodeling* (Koren et al., 1991). Although clinically correlated with adverse outcomes, this type of concentric ventricular remodeling is associated with reduced mortality compared to remodeling characterized by an elevated RWT coupled with increased cardiac mass and chamber dimensions (concentric *hypertrophy*) as observed early (2 weeks post-TAC) in FHC-TAC hearts (Koren et al., 1991; Ganau et al., 1992).

Subsequently, WT-TAC hearts follow a pathological pattern of increasing cardiac mass and chamber dimensions (dilation) defined as *eccentric hypertrophy*, whereas FHC-TAC hearts maintain chamber geometry consistent with worsening *concentric hypertrophy*. Again, patients with established concentric hypertrophy due to hypertension have the most severe clinical outcomes (Gaasch and Zile, 2011). Moreover, the increased RWT often found in individuals with valvular heart disease and/or aortic stenosis is a strong indicator for poor prognosis and is an independent risk factor for cardiovascular mortality and morbidity (Levy et al., 1990; Orsinelli et al., 1993). In patients with FHC, ventricular wall thickness is directly proportional to the clinical severity of the disease and the risk of sudden cardiac death (Spirito et al., 2000; Maron, 2002). Incidentally, LVPWd continues to thicken in FHC-TAC hearts consistent with worsening concentric hypertrophy.

Based upon hemodynamic analysis, eccentric hypertrophy in 9-week WT-TAC hearts is accompanied by elevated Ees and PRSW, indications that these hearts are in a *compensatory* state of cardiac hypertrophy with elevated cardiac function (Gaasch, 1979; Khouri et al., 2010). Likewise, the preserved ratio of Ea to Ees, an indicator of ventricular and vascular coupling, in the presence of an increased Ea, a measure of afterload, further supports this contention (Yang et al., 1999; Kass, 2002). Unlike WT hearts, cardiac function of FHC hearts following TAC demonstrates no functional increase when compared to FHC-Sham hearts, suggesting that FHC hearts respond and compensate to pressure overload differently than WT hearts. This may be due to a compensatory or pathological “limit” of FHC hearts that is different than the level attained by WT mice. Furthermore, it is conceivable that because FHC-Sham hearts demonstrate *a priori* elevated systolic parameters [Table 2 and Georgakopoulos et al. (1999)] ventricular remodeling is markedly dissimilar than WT-TAC mice and may underlie the increased mortality in FHC mice. Another finding in the present study is that gradual pressure overload increases the rate of maximal LV pressure decline (dp/dtmin) and shortens the time constant of the pressure fall (tau) in WT animals, an indication of the enhanced load dependence of myocardial relaxation (Cohn et al., 1972; Leite-Moreira and Gillebert, 1994). These responses are blunted in the FHC mutant mice. Although EDPVR, a measure of myocardial stiffness, is not different between WT and FHC mice, these data suggest a fundamental difference in ventricular properties related to relaxation. Taken together, functional evaluation and morphological assessment suggest that FHC hearts possess a limited ability to undergo compensatory hypertrophy in response to pressure overload and illustrate a different hypertrophic pattern relative to WT animals.

Considering the differential impact of TAC on morphometric and functional adaptation in WT compared to FHC mice, we shifted our focus to the cellular processes that may underlie these differences. Not surprisingly, we reveal that the FHC transgene induces a unique pattern of cellular adaptation. Using β-MyHC as a measure of pathological progression, FHC hearts show an early elevation (2 weeks) of β-MyHC compared to all other groups. WT hearts post-TAC demonstrate the typical increased expression of β-MyHC but until a later timepoint. This further indicates that FHC hearts are at a distinct pathological state at the outset. Taking into account the differences in both contractile and relaxation parameters, we targeted the temporal expression pattern of intracellular Ca^2+^-handling proteins. In addition, we evaluated the response to TAC by comparing the relative change of protein levels to the appropriate controls as opposed to comparing absolute protein levels across all experimental groups. Since the FHC transgene initiates a unique genetic program very early (Stauffer et al., 2006; Luczak et al., 2011), we reasoned that this analytical approach would be representative of adaptive potential or, more appropriately, *capacity*. Thus, we can directly test whether FHC hearts have limited *cellular* adaptive capacity at the cellular much like at the morphological and functional level detailed above. Using this strategy, we illustrate a significant decrease in SERCA2a protein levels 2 weeks post-TAC, consistent with previous models of pressure overload (Kranias and Hajjar, 2012), in WT hearts only. Furthermore, SERCA2a in WT hearts continues to fall with sustained pressure overload (9 weeks post-TAC) to levels below that of the 2-week post-TAC group compared to respective WT controls. A drop in SERCA2a protein is not seen in FHC hearts at either timepoint, again, suggesting an inability to further adapt.

PLN acts as a negative regulator of SERCA2a activity and phosphorylation of PLN relieves this inhibitory effect on SERCA2a (Kranias and Hajjar, 2012). Many studies have subsequently established a critical role for PLN expression and phosphorylation as a modulator of Ca^2+^ flux and myocyte contractility in rodent models of CHF (Kranias and Hajjar, 2012). In this study, the early response to TAC in WT hearts is to increase PLN phosphorylation at the PKA site, Ser16 (McTiernan et al., 1999), relieving the inhibition on SERCA2a and increasing Ca^2+^ uptake by the SR. This elevation of PLN-S^16^ phosphorylation in WT hearts becomes attenuated to control levels at 9 weeks post-TAC. In contrast, FHC hearts show no change in PLN protein or phosphorylation levels early but demonstrate a latent increase in PLN-S^16^ phosphorylation. It must be noted that this increase in PLN phosphorylation in partially driven by a significant decrease in PLN expression that occurs with increasing CHF progression (McTiernan et al., 1999).

### Concluding remarks

In conclusion, data from the present study demonstrates that the temporal and morphological pattern of ventricular remodeling in response to pressure overload depends not only on the presence of a mutant myosin (R403Q) transgene but also on genetic background. The differential ventricular remodeling is manifest as premature death, a more severe hypertrophy, altered load dependence of myocardial relaxation, and aberrant cellular adaption in FHC mice. Although the underlying mechanisms inducing this aberrant remodeling in the FHC mice is not known, it is most likely the combinatorial result of the pre-existing disease and the ensuing genetic and molecular signaling cascade. Given that concentric hypertrophy and diastolic dysfunction are central features of FHC and important to pathophysiology of heart failure, our findings may reflect early pathogenesis that underlies the FHC disease progression in the setting of co-existing pressure overload. Perhaps more importantly is that this study de-emphasizes the relative importance of defining the specific *genotype* as it relates to the pathophysiology and highlights the significance of identifying the mechanisms leading to the cellular *phenotype*. This latter notion is not only in line with the recent underpinnings of a statement by the NHLBI (Force et al., 2010) but also may lead to potential therapeutic targets for the prevention and treatment of FHC.

### Conflict of interest statement

The authors declare that the research was conducted in the absence of any commercial or financial relationships that could be construed as a potential conflict of interest.

## References

[B1] Assayag P. Carre F. Chevalier B. Delcayre C. Mansier P. Swynghedauw B. (1997). Compensated cardiac hypertrophy: arrhythmogenicity and the new myocardial phenotype. I. Fibrosis. Cardiovasc. Res. 34, 439–444 10.1016/S0008-6363(97)00073-4 9231026

[B2] Barrick C. J. Rojas M. Schoonhoven R. Smyth S. S. Threadgill D. W. (2007). Cardiac response to pressure overload in 129S1/SvImJ and C57BL/6J mice: temporal- and background-dependent development of concentric left ventricular hypertrophy. Am. J. Physiol. Heart Circ. Physiol. 292, H2119–H2130 10.1152/ajpheart.00816.2006 17172276

[B3] Bonne G. Carrier L. Richard P. Hainque B. Schwartz K. (1998). Familial hypertrophic cardiomyopathy: from mutations to functional defects. Circ. Res. 83, 580–593 10.1161/01.RES.83.6.580 9742053

[B4] Burkhoff D. Mirsky I. Suga H. (2005). Assessment of systolic and diastolic ventricular properties via pressure-volume analysis: a guide for clinical, translational, and basic researchers. Am. J. Physiol. Heart Circ. Physiol. 289, H501–H512 10.1152/ajpheart.00138.2005 16014610

[B5] Choi D. J. Koch W. J. Hunter J. J. Rockman H. A. (1997). Mechanism of beta-adrenergic receptor desensitization in cardiac hypertrophy is increased beta-adrenergic receptor kinase. J. Biol. Chem. 272, 17223–17229 10.1074/jbc.272.27.17223 9202046

[B6] Cohn P. F. Liedtke A. J. Serur J. Sonnenblick E. H. Urschel C. W. (1972). Maximal rate of pressure fall (peak negative dP-dt) during ventricular relaxation. Cardiovasc. Res. 6, 263–267 10.1093/cvr/6.3.263 5035596

[B7] Collins R. Peto R. MacMahon S. Hebert P. Fiebach N. H. Eberlein K. A. (1990). Blood pressure, stroke, and coronary heart disease. Part 2, Short-term reductions in blood pressure: overview of randomised drug trials in their epidemiological context. Lancet 335, 827–838 10.1016/0140-6736(90)90944-Z 1969567

[B8] Devereux R. B. de Simone G. Ganau A. Roman M. J. (1994). Left ventricular hypertrophy and geometric remodeling in hypertension: stimuli, functional consequences and prognostic implications. J. Hypertens. Suppl. 12, S117–S127 7769482

[B9] Epstein H. F. Fischman D. A. Bader D. Changeux J. P. Buckhold K. Ordahl C. P. (1992). Myoblast therapy. Science 257, 738 10.1126/science.1496388 1496388

[B10] Force T. Bonow R. O. Houser S. R. Solaro R. J. Hershberger R. E. Adhikari B. (2010). Research priorities in hypertrophic cardiomyopathy: report of a Working Group of the National Heart, Lung, and Blood Institute. Circulation 122, 1130–1133 10.1161/CIRCULATIONAHA.110.950089 20837938 PMC3070356

[B11] Gaasch W. H. (1979). Left ventricular radius to wall thickness ratio. Am. J. Cardiol. 43, 1189–1194 10.1016/0002-9149(79)90152-8 155986

[B12] Gaasch W. H. Zile M. R. (2011). Left ventricular structural remodeling in health and disease: with special emphasis on volume, mass, and geometry. J. Am. Coll. Cardiol. 58, 1733–1740 10.1016/j.jacc.2011.07.022 21996383

[B13] Ganau A. Devereux R. B. Roman M. J. de Simone G. Pickering T. G. Saba P. S. (1992). Patterns of left ventricular hypertrophy and geometric remodeling in essential hypertension. J. Am. Coll. Cardiol. 19, 1550–1558 10.1016/0735-1097(92)90617-V 1534335

[B14] Geisterfer-Lowrance A. A. Christe M. Conner D. A. Ingwall J. S. Schoen F. J. Seidman C. E. (1996). A mouse model of familial hypertrophic cardiomyopathy. Science 272, 731–734 10.1126/science.272.5262.731 8614836

[B15] Geisterfer-Lowrance A. A. Kass S. Tanigawa G. Vosberg H. P. McKenna W. Seidman C. E. (1990). A molecular basis for familial hypertrophic cardiomyopathy: a beta cardiac myosin heavy chain gene missense mutation. Cell 62, 999–1006 10.1016/0092-8674(90)90274-I 1975517

[B16] Georgakopoulos D. Christe M. E. Giewat M. Seidman C. M. Seidman J. G. Kass D. A. (1999). The pathogenesis of familial hypertrophic cardiomyopathy: early and evolving effects from an alpha-cardiac myosin heavy chain missense mutation. Nat. Med. 5, 327–330 10.1038/6549 10086390

[B17] Hill J. A. Karimi M. Kutschke W. Davisson R. L. Zimmerman K. Wang Z. (2000). Cardiac hypertrophy is not a required compensatory response to short-term pressure overload. Circulation 101, 2863–2869 10.1161/01.CIR.101.24.2863 10859294

[B18] Kannel W. B. (1996). Blood pressure as a cardiovascular risk factor: prevention and treatment. JAMA 275, 1571–1576 10.1001/jama.1996.03530440051036 8622248

[B19] Kass D. A. (2002). Age-related changes in venticular-arterial coupling: pathophysiologic implications. Heart Fail. Rev. 7, 51–62 10.1023/A:1013749806227 11790922

[B20] Khouri M. G. Peshock R. M. Ayers C. R. de Lemos J. A. Drazner M. H. (2010). A 4-tiered classification of left ventricular hypertrophy based on left ventricular geometry: the Dallas heart study. Circ. Cardiovasc. Imaging 3, 164–171 10.1161/CIRCIMAGING.109.883652 20061518

[B21] Konhilas J. P. Watson P. A. Maass A. Boucek D. M. Horn T. Stauffer B. L. (2006). Exercise can prevent and reverse the severity of hypertrophic cardiomyopathy. Circ. Res. 98, 540–548 10.1161/01.RES.0000205766.97556.00 16439687

[B22] Kooij V. Saes M. Jaquet K. Zaremba R. Foster D. B. Murphy A. M. (2010). Effect of troponin I Ser23/24 phosphorylation on Ca2+-sensitivity in human myocardium depends on the phosphorylation background. J. Mol. Cell. Cardiol. 48, 954–963 10.1016/j.yjmcc.2010.01.002 20079747 PMC2854313

[B23] Koren M. J. Devereux R. B. Casale P. N. Savage D. D. Laragh J. H. (1991). Relation of left ventricular mass and geometry to morbidity and mortality in uncomplicated essential hypertension. Ann. Intern. Med. 114, 345–352 10.7326/0003-4819-114-5-345 1825164

[B24] Kranias E. G. Hajjar R. J. (2012). Modulation of cardiac contractility by the phospholamban/SERCA2a regulatome. Circ. Res. 110, 1646–1660 10.1161/CIRCRESAHA.111.259754 22679139 PMC3392125

[B25] Lechin F. van der Dijs B. Orozco B. Lechin A. E. Baez S. Lechin M. E. (1995). Plasma neurotransmitters, blood pressure, and heart rate during supine resting, orthostasis, and moderate exercise in dysthymic depressed patients. Biol. Psychiatry 37, 884–891 10.1016/0006-3223(94)00220-W 7548463

[B26] Leite-Moreira A. F. Gillebert T. C. (1994). Nonuniform course of left ventricular pressure fall and its regulation by load and contractile state. Circulation 90, 2481–2491 10.1161/01.CIR.90.5.2481 7955206

[B27] Levy D. Garrison R. J. Savage D. D. Kannel W. B. Castelli W. P. (1990). Prognostic implications of echocardiographically determined left ventricular mass in the Framingham Heart Study. N. Engl. J. Med. 322, 1561–1566 10.1056/NEJM199005313222203 2139921

[B28] Luczak E. D. Barthel K. K. Stauffer B. L. Konhilas J. P. Cheung T. H. Leinwand L. A. (2011). Remodeling the cardiac transcriptional landscape with diet. Physiol. Genomics 43, 772–780 10.1152/physiolgenomics.00237.2010 21487031 PMC3121157

[B29] Marian A. J. Roberts R. (2001). The molecular genetic basis for hypertrophic cardiomyopathy. J. Mol. Cell. Cardiol. 33, 655–670 10.1006/jmcc.2001.1340 11273720 PMC2901497

[B30] Maron B. J. (2002). Hypertrophic cardiomyopathy: a systematic review. JAMA 287, 1308–1320 10.1001/jama.287.10.1308 11886323

[B31] Mattiazzi A. Mundina-Weilenmann C. Guoxiang C. Vittone L. Kranias E. (2005). Role of phospholamban phosphorylation on Thr17 in cardiac physiological and pathological conditions. Cardiovasc. Res. 68, 366–375 10.1016/j.cardiores.2005.08.010 16226237

[B32] McConnell B. K. Moravec C. S. Morano I. Bond M. (1997). Troponin I phosphorylation in spontaneously hypertensive rat heart: effect of beta-adrenergic stimulation. Am. J. Physiol. 273, H1440–H451 9321836 10.1152/ajpheart.1997.273.3.H1440

[B33] McTiernan C. F. Frye C. S. Lemster B. H. Kinder E. A. Ogletree-Hughes M. L. Moravec C. S. (1999). The human phospholamban gene: structure and expression. J. Mol. Cell. Cardiol. 31, 679–692 10.1006/jmcc.1998.0904 10198197

[B34] Olsson M. C. Palmer B. M. Leinwand L. A. Moore R. L. (2001). Gender and aging in a transgenic mouse model of hypertrophic cardiomyopathy. Am. J. Physiol. Heart Circ. Physiol. 280, H1136–H1144 11179057 10.1152/ajpheart.2001.280.3.H1136

[B35] Ong K. L. Cheung B. M. Man Y. B. Lau C. P. Lam K. S. (2007). Prevalence, awareness, treatment, and control of hypertension among United States adults 1999-2004. Hypertension 49, 69–75 10.1161/01.HYP.0000252676.46043.18 17159087

[B36] Orsinelli D. A. Aurigemma G. P. Battista S. Krendel S. Gaasch W. H. (1993). Left ventricular hypertrophy and mortality after aortic valve replacement for aortic stenosis. A high risk subgroup identified by preoperative relative wall thickness. J. Am. Coll. Cardiol. 22, 1679–1683 10.1016/0735-1097(93)90595-R 8227838

[B37] Ortlepp J. R. Vosberg H. P. Reith S. Ohme F. Mahon N. G. Schroder D. (2002). Genetic polymorphisms in the renin-angiotensin-aldosterone system associated with expression of left ventricular hypertrophy in hypertrophic cardiomyopathy: a study of five polymorphic genes in a family with a disease causing mutation in the myosin binding protein C gene. Heart 87, 270–275 10.1136/heart.87.3.270 11847170 PMC1767035

[B38] Osterop A. P. Kofflard M. J. Sandkuijl L. A. ten Cate F. J. Krams R. Schalekamp M. A. (1998). AT1 receptor A/C1166 polymorphism contributes to cardiac hypertrophy in subjects with hypertrophic cardiomyopathy. Hypertension 32, 825–830 10.1161/01.HYP.32.5.825 9822439

[B39] Perrino C. Naga Prasad S. V. Mao L. Noma T. Yan Z. Kim H. S. (2006). Intermittent pressure overload triggers hypertrophy-independent cardiac dysfunction and vascular rarefaction. J. Clin. Invest. 116, 1547–1560 10.1172/JCI25397 16741575 PMC1464895

[B40] Porterfield J. E. Kottam A. T. Raghavan K. Escobedo D. Jenkins J. T. Larson E. R. (2009). Dynamic correction for parallel conductance, GP, and gain factor, alpha, in invasive murine left ventricular volume measurements. J. Appl. Physiol. 107, 1693–1703 10.1152/japplphysiol.91322.2008 19696357 PMC2793194

[B41] Schmitt J. P. Semsarian C. Arad M. Gannon J. Ahmad F. Duffy C. (2003). Consequences of pressure overload on sarcomere protein mutation-induced hypertrophic cardiomyopathy. Circulation 108, 1133–1138 10.1161/01.CIR.0000086469.85750.48 12925456

[B42] Spirito P. Bellone P. Harris K. M. Bernabo P. Bruzzi P. Maron B. J. (2000). Magnitude of left ventricular hypertrophy and risk of sudden death in hypertrophic cardiomyopathy. N. Engl. J. Med. 342, 1778–1785 10.1056/NEJM200006153422403 10853000

[B43] Stauffer B. L. Konhilas J. P. Luczak E. D. Leinwand L. A. (2006). Soy diet worsens heart disease in mice. J. Clin. Invest. 116, 209–216 10.1172/JCI24676 16395406 PMC1323247

[B44] Towbin J. A. (2000). Molecular genetics of hypertrophic cardiomyopathy. Curr. Cardiol. Rep. 2, 134–140 10.1007/s11886-000-0010-9 10980884

[B45] Vikstrom K. L. Factor S. M. Leinwand L. A. (1996). Mice expressing mutant myosin heavy chains are a model for familial hypertrophic cardiomyopathy. Mol. Med. 2, 556–567 8898372 PMC2230192

[B46] Vinereanu D. Florescu N. Sculthorpe N. Tweddel A. C. Stephens M. R. Fraser A. G. (2001). Differentiation between pathologic and physiologic left ventricular hypertrophy by tissue doppler assessment of long-axis function in patients with hypertrophic cardiomyopathy or systemic hypertension and in athletes. Am. J. Cardiol. 88, 53–58 10.1016/S0002-9149(01)01585-5 11423058

[B47] Yang B. Larson D. F. Watson R. (1999). Age-related left ventricular function in the mouse: analysis based on *in vivo* pressure-volume relationships. Am. J. Physiol. 277, H1906–H1913 10564146 10.1152/ajpheart.1999.277.5.H1906

